# Supplementary material for: Modeling glioblastoma heterogeneity as a dynamic network of cell states
Source: Mol Syst Biol. 2021 Sep 16;17(9):e10105. doi: 10.15252/msb.202010105 (PMC8444284; doi:10.15252/msb.202010105)
Supplement: Supplementary file 5 — Source Data for Figure 3 [file MSB-17-e10105-s001.zip › Figure3A_sourcedata/GSEA_3065/hallmarks_state4.GseaPreranked.1623416296068/HALLMARK_MYC_TARGETS_V1.html]

Details for gene set HALLMARK\_MYC\_TARGETS\_V1[GSEA]

|  || Dataset | state4 |
| Phenotype | NoPhenotypeAvailable |
| Upregulated in class | na\_neg |
| GeneSet | HALLMARK\_MYC\_TARGETS\_V1 |
| Enrichment Score (ES) | -0.5571541 |
| Normalized Enrichment Score (NES) | -2.1614933 |
| Nominal p-value | 0.0 |
| FDR q-value | 0.0 |
| FWER p-Value | 0.0 |
Table: GSEA Results Summary

  

Fig 1: Enrichment plot: HALLMARK\_MYC\_TARGETS\_V1      
 Profile of the Running ES Score & Positions of GeneSet Members on the Rank Ordered List

  

| PROBE | GENE SYMBOL | GENE\_TITLE | RANK IN GENE LIST | RANK METRIC SCORE | RUNNING ES | CORE ENRICHMENT || 1 | RPL34 |  |  | 69 | 0.278 | 0.0017 | No |
| 2 | RPL6 |  |  | 142 | 0.225 | 0.0005 | No |
| 3 | UBE2E1 |  |  | 342 | 0.167 | -0.0243 | No |
| 4 | RPS6 |  |  | 343 | 0.166 | -0.0165 | No |
| 5 | RPS5 |  |  | 427 | 0.147 | -0.0232 | No |
| 6 | RPL22 |  |  | 473 | 0.141 | -0.0240 | No |
| 7 | RACK1 |  |  | 479 | 0.140 | -0.0182 | No |
| 8 | RPL18 |  |  | 549 | 0.131 | -0.0234 | No |
| 9 | RPS2 |  |  | 711 | 0.113 | -0.0445 | No |
| 10 | YWHAE |  |  | 724 | 0.111 | -0.0413 | No |
| 11 | RPLP0 |  |  | 757 | 0.108 | -0.0415 | No |
| 12 | SNRPD2 |  |  | 801 | 0.104 | -0.0437 | No |
| 13 | RPS10 |  |  | 862 | 0.099 | -0.0489 | No |
| 14 | CLNS1A |  |  | 984 | 0.089 | -0.0646 | No |
| 15 | HDDC2 |  |  | 999 | 0.088 | -0.0627 | No |
| 16 | RPS3 |  |  | 1098 | 0.082 | -0.0750 | No |
| 17 | PABPC1 |  |  | 1104 | 0.082 | -0.0719 | No |
| 18 | EIF4G2 |  |  | 1178 | 0.077 | -0.0803 | No |
| 19 | YWHAQ |  |  | 1251 | 0.073 | -0.0887 | No |
| 20 | RPL14 |  |  | 1287 | 0.071 | -0.0910 | No |
| 21 | MRPS18B |  |  | 1348 | 0.069 | -0.0977 | No |
| 22 | TXNL4A |  |  | 1523 | 0.061 | -0.1234 | No |
| 23 | NCBP2 |  |  | 1727 | 0.055 | -0.1541 | No |
| 24 | NDUFAB1 |  |  | 1773 | 0.053 | -0.1590 | No |
| 25 | AP3S1 |  |  | 1884 | 0.050 | -0.1747 | No |
| 26 | LSM7 |  |  | 1999 | 0.047 | -0.1913 | No |
| 27 | FAM120A |  |  | 2020 | 0.046 | -0.1924 | No |
| 28 | APEX1 |  |  | 2159 | 0.042 | -0.2130 | No |
| 29 | PCBP1 |  |  | 2226 | 0.041 | -0.2219 | No |
| 30 | NHP2 |  |  | 2254 | 0.040 | -0.2245 | No |
| 31 | PRPF31 |  |  | 2317 | 0.039 | -0.2328 | No |
| 32 | EIF3D |  |  | 2502 | 0.035 | -0.2614 | No |
| 33 | SRM |  |  | 2657 | 0.032 | -0.2852 | No |
| 34 | PSMB3 |  |  | 2696 | 0.031 | -0.2900 | No |
| 35 | PSMD7 |  |  | 2730 | 0.030 | -0.2940 | No |
| 36 | UBE2L3 |  |  | 2763 | 0.029 | -0.2979 | No |
| 37 | PPIA |  |  | 2777 | 0.029 | -0.2987 | No |
| 38 | CNBP |  |  | 2838 | 0.028 | -0.3072 | No |
| 39 | NAP1L1 |  |  | 2856 | 0.027 | -0.3087 | No |
| 40 | MRPL23 |  |  | 2947 | 0.026 | -0.3223 | No |
| 41 | RAD23B |  |  | 2951 | 0.026 | -0.3216 | No |
| 42 | PGK1 |  |  | 3012 | 0.025 | -0.3303 | No |
| 43 | STARD7 |  |  | 3083 | 0.023 | -0.3407 | No |
| 44 | PHB2 |  |  | 3200 | 0.021 | -0.3587 | No |
| 45 | SLC25A3 |  |  | 3260 | 0.020 | -0.3675 | No |
| 46 | PRDX3 |  |  | 3387 | 0.016 | -0.3874 | No |
| 47 | CDK4 |  |  | 3394 | 0.016 | -0.3877 | No |
| 48 | HDAC2 |  |  | 3407 | 0.015 | -0.3889 | No |
| 49 | SSB |  |  | 3565 | 0.010 | -0.4142 | No |
| 50 | PSMD8 |  |  | 3602 | 0.009 | -0.4197 | No |
| 51 | SNRPB2 |  |  | 3647 | 0.007 | -0.4266 | No |
| 52 | EIF4E |  |  | 3810 | 0.000 | -0.4532 | No |
| 53 | CSTF2 |  |  | 3811 | 0.000 | -0.4532 | No |
| 54 | PWP1 |  |  | 3853 | -0.002 | -0.4598 | No |
| 55 | COPS5 |  |  | 3885 | -0.003 | -0.4648 | No |
| 56 | ERH |  |  | 3933 | -0.006 | -0.4722 | No |
| 57 | NOP16 |  |  | 3977 | -0.008 | -0.4789 | No |
| 58 | HNRNPC |  |  | 3981 | -0.008 | -0.4790 | No |
| 59 | VBP1 |  |  | 3996 | -0.009 | -0.4808 | No |
| 60 | COX5A |  |  | 4019 | -0.010 | -0.4840 | No |
| 61 | EXOSC7 |  |  | 4026 | -0.011 | -0.4844 | No |
| 62 | HNRNPA1 |  |  | 4032 | -0.011 | -0.4847 | No |
| 63 | PSMA1 |  |  | 4157 | -0.019 | -0.5042 | No |
| 64 | PSMD3 |  |  | 4201 | -0.022 | -0.5102 | No |
| 65 | PSMC4 |  |  | 4249 | -0.025 | -0.5168 | No |
| 66 | EIF2S2 |  |  | 4252 | -0.025 | -0.5159 | No |
| 67 | ACP1 |  |  | 4266 | -0.026 | -0.5168 | No |
| 68 | PSMA2 |  |  | 4300 | -0.029 | -0.5209 | No |
| 69 | GSPT1 |  |  | 4383 | -0.034 | -0.5327 | No |
| 70 | TFDP1 |  |  | 4389 | -0.035 | -0.5319 | No |
| 71 | RSL1D1 |  |  | 4399 | -0.036 | -0.5317 | No |
| 72 | XRCC6 |  |  | 4437 | -0.039 | -0.5360 | No |
| 73 | LSM2 |  |  | 4506 | -0.045 | -0.5450 | No |
| 74 | PSMB2 |  |  | 4557 | -0.048 | -0.5510 | No |
| 75 | PHB |  |  | 4584 | -0.050 | -0.5529 | No |
| 76 | EIF4H |  |  | 4611 | -0.052 | -0.5547 | Yes |
| 77 | MYC |  |  | 4618 | -0.053 | -0.5532 | Yes |
| 78 | GLO1 |  |  | 4640 | -0.054 | -0.5541 | Yes |
| 79 | SERBP1 |  |  | 4641 | -0.054 | -0.5516 | Yes |
| 80 | CYC1 |  |  | 4648 | -0.055 | -0.5500 | Yes |
| 81 | PSMA7 |  |  | 4651 | -0.055 | -0.5477 | Yes |
| 82 | TRIM28 |  |  | 4656 | -0.056 | -0.5457 | Yes |
| 83 | IFRD1 |  |  | 4671 | -0.057 | -0.5453 | Yes |
| 84 | PRPS2 |  |  | 4693 | -0.059 | -0.5460 | Yes |
| 85 | EIF1AX |  |  | 4700 | -0.060 | -0.5442 | Yes |
| 86 | GNL3 |  |  | 4704 | -0.060 | -0.5419 | Yes |
| 87 | POLD2 |  |  | 4714 | -0.061 | -0.5405 | Yes |
| 88 | HDGF |  |  | 4737 | -0.063 | -0.5412 | Yes |
| 89 | HSPE1 |  |  | 4744 | -0.063 | -0.5392 | Yes |
| 90 | VDAC1 |  |  | 4748 | -0.064 | -0.5367 | Yes |
| 91 | SSBP1 |  |  | 4804 | -0.068 | -0.5425 | Yes |
| 92 | SNRPD3 |  |  | 4815 | -0.069 | -0.5409 | Yes |
| 93 | IMPDH2 |  |  | 4827 | -0.070 | -0.5394 | Yes |
| 94 | PSMC6 |  |  | 4872 | -0.073 | -0.5432 | Yes |
| 95 | SYNCRIP |  |  | 4908 | -0.075 | -0.5454 | Yes |
| 96 | HNRNPR |  |  | 4924 | -0.077 | -0.5443 | Yes |
| 97 | EIF3B |  |  | 4935 | -0.078 | -0.5423 | Yes |
| 98 | SMARCC1 |  |  | 4941 | -0.078 | -0.5394 | Yes |
| 99 | PSMD1 |  |  | 4957 | -0.079 | -0.5381 | Yes |
| 100 | CCT7 |  |  | 4968 | -0.080 | -0.5360 | Yes |
| 101 | VDAC3 |  |  | 4979 | -0.081 | -0.5338 | Yes |
| 102 | PSMA4 |  |  | 4981 | -0.081 | -0.5302 | Yes |
| 103 | SET |  |  | 4997 | -0.082 | -0.5288 | Yes |
| 104 | SNRPA |  |  | 5009 | -0.083 | -0.5267 | Yes |
| 105 | CDK2 |  |  | 5015 | -0.084 | -0.5236 | Yes |
| 106 | NME1 |  |  | 5086 | -0.090 | -0.5308 | Yes |
| 107 | C1QBP |  |  | 5104 | -0.092 | -0.5293 | Yes |
| 108 | EEF1B2 |  |  | 5106 | -0.092 | -0.5251 | Yes |
| 109 | DDX18 |  |  | 5207 | -0.102 | -0.5367 | Yes |
| 110 | PPM1G |  |  | 5229 | -0.104 | -0.5353 | Yes |
| 111 | SRSF1 |  |  | 5281 | -0.108 | -0.5386 | Yes |
| 112 | UBA2 |  |  | 5328 | -0.111 | -0.5409 | Yes |
| 113 | CCT3 |  |  | 5330 | -0.112 | -0.5358 | Yes |
| 114 | ILF2 |  |  | 5331 | -0.112 | -0.5306 | Yes |
| 115 | SRPK1 |  |  | 5360 | -0.115 | -0.5298 | Yes |
| 116 | TCP1 |  |  | 5367 | -0.115 | -0.5254 | Yes |
| 117 | CBX3 |  |  | 5377 | -0.116 | -0.5214 | Yes |
| 118 | PABPC4 |  |  | 5447 | -0.124 | -0.5269 | Yes |
| 119 | HNRNPA3 |  |  | 5455 | -0.126 | -0.5221 | Yes |
| 120 | PSMD14 |  |  | 5460 | -0.126 | -0.5168 | Yes |
| 121 | CCT4 |  |  | 5474 | -0.128 | -0.5129 | Yes |
| 122 | POLE3 |  |  | 5481 | -0.129 | -0.5078 | Yes |
| 123 | EIF4A1 |  |  | 5506 | -0.132 | -0.5056 | Yes |
| 124 | KPNB1 |  |  | 5514 | -0.133 | -0.5005 | Yes |
| 125 | SNRPG |  |  | 5530 | -0.134 | -0.4966 | Yes |
| 126 | HPRT1 |  |  | 5554 | -0.136 | -0.4940 | Yes |
| 127 | BUB3 |  |  | 5583 | -0.141 | -0.4920 | Yes |
| 128 | ABCE1 |  |  | 5606 | -0.145 | -0.4888 | Yes |
| 129 | G3BP1 |  |  | 5627 | -0.148 | -0.4851 | Yes |
| 130 | CTPS1 |  |  | 5635 | -0.150 | -0.4792 | Yes |
| 131 | MCM6 |  |  | 5644 | -0.151 | -0.4734 | Yes |
| 132 | TRA2B |  |  | 5652 | -0.152 | -0.4674 | Yes |
| 133 | CCT2 |  |  | 5654 | -0.152 | -0.4604 | Yes |
| 134 | TUFM |  |  | 5675 | -0.156 | -0.4564 | Yes |
| 135 | RNPS1 |  |  | 5685 | -0.157 | -0.4504 | Yes |
| 136 | HNRNPD |  |  | 5705 | -0.161 | -0.4460 | Yes |
| 137 | CANX |  |  | 5755 | -0.173 | -0.4459 | Yes |
| 138 | SNRPA1 |  |  | 5780 | -0.179 | -0.4414 | Yes |
| 139 | MCM2 |  |  | 5785 | -0.180 | -0.4336 | Yes |
| 140 | PA2G4 |  |  | 5841 | -0.193 | -0.4336 | Yes |
| 141 | NPM1 |  |  | 5884 | -0.203 | -0.4309 | Yes |
| 142 | SNRPD1 |  |  | 5911 | -0.210 | -0.4254 | Yes |
| 143 | PTGES3 |  |  | 5912 | -0.210 | -0.4155 | Yes |
| 144 | NOP56 |  |  | 5921 | -0.213 | -0.4068 | Yes |
| 145 | RANBP1 |  |  | 5939 | -0.218 | -0.3994 | Yes |
| 146 | XPO1 |  |  | 5964 | -0.227 | -0.3927 | Yes |
| 147 | HSPD1 |  |  | 5967 | -0.227 | -0.3823 | Yes |
| 148 | SRSF3 |  |  | 5969 | -0.227 | -0.3718 | Yes |
| 149 | USP1 |  |  | 5986 | -0.233 | -0.3635 | Yes |
| 150 | RAN |  |  | 5990 | -0.235 | -0.3529 | Yes |
| 151 | HNRNPU |  |  | 6008 | -0.241 | -0.3444 | Yes |
| 152 | HNRNPA2B1 |  |  | 6029 | -0.251 | -0.3359 | Yes |
| 153 | LDHA |  |  | 6057 | -0.272 | -0.3276 | Yes |
| 154 | CDC45 |  |  | 6063 | -0.276 | -0.3154 | Yes |
| 155 | MCM5 |  |  | 6069 | -0.278 | -0.3032 | Yes |
| 156 | DEK |  |  | 6092 | -0.292 | -0.2931 | Yes |
| 157 | CCNA2 |  |  | 6100 | -0.297 | -0.2803 | Yes |
| 158 | DUT |  |  | 6107 | -0.303 | -0.2670 | Yes |
| 159 | CCT5 |  |  | 6116 | -0.312 | -0.2537 | Yes |
| 160 | HSP90AB1 |  |  | 6119 | -0.312 | -0.2393 | Yes |
| 161 | SRSF2 |  |  | 6128 | -0.321 | -0.2256 | Yes |
| 162 | RRM1 |  |  | 6147 | -0.338 | -0.2126 | Yes |
| 163 | SRSF7 |  |  | 6150 | -0.343 | -0.1968 | Yes |
| 164 | CDC20 |  |  | 6154 | -0.344 | -0.1812 | Yes |
| 165 | PRDX4 |  |  | 6155 | -0.345 | -0.1650 | Yes |
| 166 | MCM4 |  |  | 6171 | -0.372 | -0.1499 | Yes |
| 167 | PCNA |  |  | 6173 | -0.374 | -0.1326 | Yes |
| 168 | ODC1 |  |  | 6175 | -0.377 | -0.1150 | Yes |
| 169 | RFC4 |  |  | 6185 | -0.399 | -0.0977 | Yes |
| 170 | TYMS |  |  | 6222 | -0.501 | -0.0801 | Yes |
| 171 | KPNA2 |  |  | 6236 | -0.543 | -0.0567 | Yes |
| 172 | MAD2L1 |  |  | 6247 | -0.628 | -0.0288 | Yes |
| 173 | MCM7 |  |  | 6252 | -0.665 | 0.0018 | Yes |
Table: GSEA details [plain text format]

  

Fig 2: HALLMARK\_MYC\_TARGETS\_V1: Random ES distribution      
 Gene set null distribution of ES for **HALLMARK\_MYC\_TARGETS\_V1**
